# Cross-sectional study on prevalence and risk factors of undiagnosed type 2 diabetes among patients with hypertension attending St. Orsola Catholic Mission Hospital, Tharaka Nithi County, Kenya

**DOI:** 10.11604/pamj.2024.49.41.44119

**Published:** 2024-10-15

**Authors:** Gerrald Njeruh Emilioh, Josephat Nyagero, Rumishael Shoo

**Affiliations:** 1 Department of Community Health, School of Public Health, Amref International University, Nairobi, Kenya

**Keywords:** Type 2 diabetes, hypertension, diabetes medication, diabetes

## Abstract

**Introduction:**

according to the World Health Organization (WHO), Non-Communicable Diseases (NCD) were a major cause of death in 2022 accounting for 4 million (74%) of deaths worldwide. Diabetes mellitus and hypertension are the two illnesses that are not contagious but linked closely. The objective of the research was to establish the prevalence and risk factors of undiagnosed diabetes among patients with hypertension attending St. Orsola Hospital in Tharaka Nithi County, Kenya.

**Methods:**

the study utilized a descriptive cross-sectional design with random sampling. Data were collected from 384 patients with hypertension attending outpatient medical from October to December 2022 using a structured questionnaire. Analysis was conducted using the Statistical Package for the Social Sciences (SPSS). The chi-square test was used at the bivariable level and multiple logistic regression at the multivariable level, with a significance level set at P<0.05.

**Results:**

the findings revealed that the age of the participants ranged between 20-89 years, with majority (62%) being below 60 years, where of these participants (66%) were women. Seventy-five percent (288/384) of participants were found to be with no diabetes, with 21 (5.5%) with undiagnosed diabetes mellitus and 75 (19.5%) being pre-diabetes. Significant associations were found between diabetes status and socio-demographic factors, with higher body mass index (BMI > 24.9) showing a strong correlation with undiagnosed diabetes (AOR 3.794 95% CI: 1.345-4.705). Education level was also significant, with lower education levels (primary or below) associated with a higher risk of undiagnosed diabetes (AOR 1.821 95% CI: 2.134-8.567). Employment status played a critical role, with unemployed individuals more likely to have undiagnosed diabetes (AOR 2.845 95% CI: 1.211-6.683). Additionally, lower frequency of vegetable consumption (less than three times per week) was linked to a higher likelihood of undiagnosed diabetes (AOR 2.937 95% CI: 1.135-7.602). Gender disparities were evident, with 62% of undiagnosed diabetes cases occurring in women. These findings underscore the importance of addressing both socio-economic and behavioral factors in the prevention and management of undiagnosed diabetes among patients with hypertension.

**Conclusion:**

the study highlights a substantial prevalence of undiagnosed diabetes among patients with hypertension. These findings underscore the need for integrated screening programs, targeted health education, and lifestyle modification interventions.

## Introduction

The most common non-communicable diseases worldwide are diabetes and hypertension, which together seriously compromise health. These disorders raise your risk of kidney disease, heart attacks, and vision problems [1]. Particularly vulnerable are hypertension patients with undiagnosed type 2 diabetes, thus early diagnosis and treatment are absolutely vital [2]. Rising arterial blood pressure, marks the hallmark of hypertension, which results from underlying medical disorders, lifestyle choices, and genetics. Complications including heart attacks, strokes, kidney disease, and vision loss follow from this over time. According to [3], with the highest frequency in Africa, almost 1.13 billion people globally suffer from hypertension; this condition affects 27% of people over 25. Along with blood pressure medication, treatment consists of lifestyle modifications including cutting salt intake and boosting exercise [4]. Influenced by obesity, inactivity, and poor diet, type 2 diabetes greatly adds to the worldwide disease load. Unlike autoimmune type 1 diabetes, which requires insulin and usually strikes in childhood or adolescence, type 2 diabetes can be controlled with lifestyle changes and medication [5]. Untreated, however, results in major complications including kidney disease, heart disease, nerve damage, and blindness [6].

Diabetes and hypertension are risk factors for cardiovascular problems, thus they are strongly linked [7]. Early management of these diseases depends critically on prevention strategies including a diet high in fruits and whole grains, regular exercise, and a good weight; also important are frequent visits and tests. Type 2 diabetes and hypertension often coexist. Cardiovascular and microvascular complications increase with coexisting conditions. Managing and treating this coexistence requires understanding its causes. Comprehensive diabetes and hypertension management must address related factors. Regular exercise, a healthy diet, and quitting smoking prevent and manage both conditions [8-10] Focusing on optimizing blood pressure and glycaemic control to lower complications, effective management of these diseases entails frequent exercise, a healthy diet, and smoking cessation. Timeliness in treatment and better health outcomes for patients with both hypertension and diabetes depend on early screening and diagnosis using tests including fasting blood sugar, oral glucose tolerance, and HbA1c [10,11]. Hence it is against the background of the study, that this study aimed to fill the knowledge gap on the prevalence and risk factors of undiagnosed type 2 diabetes among patients with hypertension attending St. Orsola Catholic Mission Hospital, Tharaka Nithi County, Kenya.

## Methods

**Study design and setting:** this study employed a cross-sectional design to investigate the prevalence and risk factors of undiagnosed type 2 diabetes, this is because this approach, researchers to analyze data from a population at a single point in time, providing a snapshot of the prevalence and relationships between variables without the need for long-term follow-up.

**Study population:** target population refers to the whole collection of people or organizations a researcher wants to investigate and draw generalizations about. The study population (patients with hypertension) was sampled through systemic random sampling (every 2^nd^ patient was sampled). The study population was patients with hypertension (patients on anti-hypertensive medication) attending St. Orsola Catholic Mission Hospital were the target population. The occurrence of diabetes mellitus (tested by RBS and confirmed by HbA1c test) among patients with hypertension attending St. Orsola Catholic Mission Hospital was the dependent variable. Independent variables: socio-economic characteristics (age, sex, education, marital status, occupational): behavioral factors (hypertension, smoking, BMI, Nutritional status). Respondents to this study were identified following the inclusion criteria below. The patients who are already under anti-hypertensive medication or confirmed as hypertensive were included in this study. Exclusion criteria; the patients with hypertension who were known to be diabetic or under anti-diabetes medication during the study period were excluded from the study. Expectant mothers due to gestation-related diabetes and hypertension (HTN) which may disappear after delivery. The formula below was used to get the sample size (Fisher’s formula):


$$\begin{array}{l} N=\frac{{\left(z\right)}^{2}p\left(1-p\right)}{d^{2}}\\ 384=\frac{{\left(1.96\right)}^{2}\times 0.5\left(1-0.5\right)}{0.05^{2}} \end{array}$$

Where, n= desired sample size; z= standard normal distribution value (1.96), p= known prevalence rate for the factor of interest under study (50%) d= the level of desired precision (0.05). A prevalence of 50% will be used as the assumed prevalence of undiagnosed diabetics´ state among patients with hypertension d= 0.05, z= 1.96 p= 0.5, n=384.

**Collection:** patients who were already under anti-hypertensive medication or confirmed as hypertensive were given semi-structured questionnaires to fill. The researcher gave the participants one hour to fill in the questionnaires and later collected them. Bivariable and multivariable were used to show the relationship between variables.

**Laboratory analysis:** in assessing the occurrence of diabetes mellitus among patients with hypertension at St. Orsola Catholic Mission Hospital, the lab tests utilized include random blood sugar (RBS) and hemoglobin A1c (HbA1c) tests. Diabetes mellitus, the dependent variable in this study, is confirmed when a patient's fasting blood glucose level exceeds 7.0 mmol/l or when the HbA1c value is above 6.5%, in accordance with WHO definitions. The RBS test provides an initial indication of elevated blood sugar levels, which is further validated by the HbA1c test that reflects average blood glucose levels over the past two to three months, ensuring a more accurate diagnosis of diabetes in the hypertensive patient population.

### Definitions

**Diabetes mellitus:** this is a metabolic disorder characterized by high blood sugar levels, resulting from the body's inability to produce or effectively use insulin, a hormone that regulates blood sugar. The WHO currently defines it as a fasting blood glucose level over 7.0 mmol/l, or a glycated hemoglobin A1C above 6.5%.

### Socio-economic characteristics

**Age:** the number of years from birth to the time of the study.

**Sex:** the biological distinction between male and female participants.

**Education:** the highest level of formal education attained by the participant.

**Marital status:** the participant's current legal relationship status, such as single, married, divorced, or widowed.

**Occupation:** the primary type of work or employment status of the participant, categorized into different sectors or types of jobs.

### Behavioural factors

**Hypertension:** the presence of high blood pressure, confirmed by medical diagnosis or measurement during the study.

**Smoking:** the participant's current or past use of tobacco products, categorized as a smoker, non-smoker, or former smoker.

**Body mass index:** a numerical value derived from the participant's weight and height, used to classify underweight, normal weight, overweight, and obesity status.

**Nutritional status:** the participant's dietary intake and nutritional habits, are assessed through dietary surveys or indicators like malnutrition or balanced diet adherence.

**Statistical analysis:** the analysis of data was done using SPSS Version 20.0 which is a statistical software for statistical solutions, where bivariable and multivariable analysis were done to show the relationship between the variables, and look at statistical significance at a p-value less than 0.05 at a 95% confidence interval. Interpretation of the data analysis was presented in frequencies, percentages, means and standard deviation. They were presented in tables.

**Ethical considerations:** the study received endorsements from the Ethical Review Committee of Amref International University with serial number ESRC P1174/2022, whereby the researcher used it to apply for the National Commission For Science, Technology and Innovation Headquarters (NACOSTI) permit which was used to present it to the officials of St. Orsola Catholic Mission Hospital for approval of data collection from their patients.

## Results

**Demographic information of the participants:** the study sought to establish the diabetes status among the respondents. Three-quarters of the respondents 75% were non-diabetes while 19.5% were pre-diabetes but 5.5% were found to be diabetes as shown in Table 1.

**Table 1:** descriptive statistics of the respondent

Age	Frequency	Percent
<30	77	20
30 and above	307	60
**Gender**		
Male	169	44
Female	215	66
**Education level**	Frequency	Percent
Primary level and below	253	66
Secondary level and above	131	34
**Ethnicity**	Frequency	Percent
Central Ethnicity	322	84
Others	62	16
**Marital status**	Frequency	Percent
Married	242	63
Single	29	37
**Employment status**	Frequency	Percent
Employed	159	41.4
Un-employed	225	58.6
**Smoking**	Frequency	Percent
Yes	76	20
No	308	80
**Alcohol consumption**	Frequency	Percent
Yes	138	35.9
No	246	64.1
**Type cooking oil**	Frequency	Percent
Others	115	4
Liquid	269	70
Vegetables consumption/week number of days in a week	Frequency	Percent
3 and below	334	86
Above 3	50	14
Vegetables consumption/week	Frequency	Percent
3 days or less per week	315	82
More than 3	69	15
**Diabetes status**	Frequency	Percent
Non-diabetic	288	75.00
Pre-diabetic	75	19.50
Diabetic	21	5.50

**Socio-demographics of the participants:** from Table 1, the majority of the respondents (66%) were female, the majority of the respondents (80%) were aged between 30 and above years, majority of the respondents 65.8% had either primary or less than the primary level of education. The marital status of the participants shows that a majority (63%) of the respondents were married. The research sought to establish the employment status of the respondents. The highest population (41.4%) of the respondents were employed.

**Behavioral characteristics of the participants:** a majority (80%) of the respondents were non-smokers. Table 1 sought to find out whether the respondents consumed alcohol at the time of carrying out the study. A majority (64.1%) of the respondents had never consumed alcohol. Table 1 sought to find out the type of oil that the respondents used for cooking. A majority of the respondents (70%) used liquid oil. The study sought to establish how frequently the respondents consumed fruits. Table 1 indicates the average number of days during which they consumed fruits weekly. Most respondents (82%) consumed fruits for 3 days weekly, and the frequency of their vegetable consumption was weekly. The highest proportion of the respondents (86%) indicated that they consumed vegetables for an average of 3 days weekly.

**Bivariable and multivariable analysis:** bivariable analysis was conducted for all variables that showed significant association at the Chi-square level and occurrence of diabetes among patients with hypertension and other patients as shown in Table 2. Among the socio-demographic characteristics examined, a statistically significant association was observed for education level, employment status, age, and marital status, each at a significance of p=0.037, 0.029, 0.000, and 0.017 respectively. Consumption of fruits, consumption of vegetables, type of oil used, and BMI of (p=0.032, 0.005 0.044, and 0.000) respectively were the behavioral characteristics that had a statistically significant association with the occurrence of diabetes among patients with hypertension. Multivariable analysis was also done to establish relationships between the occurrence of diabetes among patients with hypertension and other variables examined. The variables with statistically significant associations are shown in Table 2. Results of the regression analysis mirror those of the bivariable analysis, indicating statistically significant associations between occurrence of diabetes among patients with hypertension and employment status (AOR 2.845 95% CI: 1.211-6.683), BMI more than 24.9 (AOR 3.794 95% CI: 1.345-4.705), education level of primary level and above (AOR 1.821 95% CI: 2.134-8.567) and consumption of vegetable more than three times per week (AOR 2.937,95% CI: 1.135-7.602).

**Table 2:** association between occurrence of diabetes status among patients with hypertension and other variables

	Occurrence of diabetes among patients with hypertension			
Variable	Unadjusted ORs (95% CI)	P-value	Adjusted ORs (95% CI)	P-value
**Education level**				
Primary level and below	1.81 (1.40-2.35)	0.001	1.821 (2.134-8.567)	0.001
**Marital status**				
Single	1.80 (0.92-1.32)	0.309	2.9 (0.565-1.531)	0.2
**Employment status**				
Unemployed	2.99 (2.35-3.82)	0.001	2.845 (1.211-6.683)	0.016
**Body mass index status**				
Body mass index >24.9	2.1(1.143-4.508)	0.036	3.794 (1.345-4.705)	0.012
**Vegetable consumption**				
< 3 times per week	2.3(1.213-1.305)	0.027	2.937 (1.135-7.602)	0.026
**Fruits consumption**				
< 3 times per week	2.775 (1.057-35.8)	0.018	0.97 (0.81-1.16)	0.731
**Types of cooking fat**				
Others	0.7 (0.020-0.168	0.045	0.6 (0.289-1.116)	0.101

## Discussion

The goal of the research was to determine the prevalence and associated risk factors for undiagnosed diabetes among individuals with hypertension at St. Orsola Hospital. This study highlights the significant prevalence of diabetes and pre-diabetes among the surveyed population, with 5.5% of respondents diagnosed with diabetes and 19.5% identified as pre-diabetic. The literature supports the finding that patients with cardiovascular diseases, such as hypertension, are more likely to have an abnormal glucose regulation (AGR). For instance, a study conducted in Uganda with 320 hypertension patients found that half of the participants were pre-diabetic, while 77 (24%) had undetected diabetes. Similarly, in Nigeria, screening 320 hypertensive individuals revealed that 105 (33%) had undetected diabetes mellitus [12]. Another study in Minnesota showed that of 3,847 hypertensive individuals, 19.6% had undiagnosed diabetes, and 10.7% had pre-diabetes [13]. The Euro heart study on diabetes mellitus and the cardiovascular system further corroborates these findings, showing that patients with coronary artery diseases and hypertension often have abnormal AGR, with 36% of participants being pre-diabetic and 22% having diabetes mellitus [14]. These findings collectively emphasize the urgent need for monitoring diabetes mellitus in all patients with hypertension in a clinical setting to enable early management [15].

In this study, a majority (66%) of the participants were women, while men constituted 34%. Similar research conducted in Uganda at Mulago Hospital found that 73% of hypertensive individuals with undetected diabetes mellitus were women [16]. This gender disparity can be largely attributed to age-related changes in the body, such as decreased insulin sensitivity and impaired glucose regulation, which become more pronounced after the age of 45. The prevalence of type 2 diabetes, which accounts for the majority of diabetes cases worldwide, is strongly associated with age. As individuals age, their risk of developing type 2 diabetes increases due to factors such as poor diet, sedentary behavior, weight gain [17], reduced insulin sensitivity, and changes in body composition, including increased body fat and decreased muscle mass. Additionally, the pancreas may become less efficient at producing insulin, further impairing glucose control and increasing the risk of diabetes [18]. The multivariable analysis in this study identified a statistically significant association between diabetes occurrence and hypertension among patients. It also highlighted several socio-demographic factors linked to the prevalence of diabetes and pre-diabetes, consistent with previous research. Education and employment status were significant predictors of diabetes at the multivariable level. These factors are well-documented in the literature, where lower educational attainment and unstable employment are recognized as key risk factors for diabetes [19,20]. Individuals with lower education levels often have limited access to health-promoting resources and are less likely to engage in preventive healthcare behaviors, increasing their risk of developing diabetes [21]. Additionally, lower education is correlated with poorer health literacy, which can hinder the effective management of chronic conditions like diabetes [22].

Employment instability was also significantly associated with diabetes. It can lead to financial insecurity, which may restrict access to healthy foods and healthcare, thereby heightening the risk of diabetes [23]. Moreover, the stress from job instability can elevate cortisol levels, reducing insulin sensitivity and increasing the likelihood of developing type 2 diabetes. Behavioral factors, including BMI, dietary habits, and the type of fat used in cooking, were significantly associated with undiagnosed diabetes. The multivariate analysis showed that a higher BMI was a significant predictor of undiagnosed diabetes, aligning with existing literature that identifies obesity as a major risk factor for type 2 diabetes [24,25]. The relationship between obesity and diabetes is well-documented, with excess adipose tissue contributing to insulin resistance, a primary mechanism in the development of type 2 diabetes [26]. Additionally, studies have shown that abdominal obesity, in particular, is more strongly associated with the risk of developing diabetes compared to overall obesity [27]. Dietary habits, specifically the frequency of vegetable consumption, also played a crucial role in the prevalence of undiagnosed diabetes. The study found that less frequent consumption of vegetables was significantly associated with undiagnosed diabetes. This is consistent with research indicating that diets rich in vegetables, which are high in dietary fibre, vitamins, and antioxidants, are protective against the development of diabetes [14,20].

Regular consumption of fruits and vegetables has been associated with improved insulin sensitivity and lower inflammation levels, both of which are important for reducing diabetes risk [28]. Additionally, the type of fat used in cooking was identified as a behavioral factor associated with diabetes at the bivariable level. Studies suggest that diets high in saturated fats, as opposed to unsaturated fats, can contribute to insulin resistance and an increased risk of type 2 diabetes [29]. Replacing saturated fats with healthier fats, such as those found in olive oil or nuts, has been shown to reduce diabetes risk by improving lipid profiles and reducing inflammation [30]. One of the study's strengths is its clear focus on a specific population of 384 patients with hypertension, which allows for a thorough analysis within this group. However, the sample size and scope do present limitations, as the findings may not be generalizable to the larger population. Additionally, the exclusion of pregnant women and those already diagnosed with diabetes may have left out important information. The lack of notable results on behavioral elements also points to the need for further research to understand their contribution to the development of diabetes better.

## Conclusion

The co-occurrence of hypertension and diabetes presents a complex and multifaceted challenge. While current challenges include limited awareness, health disparities, complex treatment regimens, and healthcare system limitations, future directions should focus on preventive strategies, integrated care models, telehealth solutions, patient-centred care, community health initiatives, improved health literacy, research and innovation, and health equity initiatives. By addressing these challenges and embracing these future directions, healthcare systems and public health organizations can provide more effective care and support for individuals facing the co-occurrence of hypertension and diabetes, ultimately improving patient outcomes and reducing the overall public health burden Preventive measures play a crucial role in managing these conditions effectively. Lifestyle modifications such as regular exercise, maintaining a healthy weight, adopting a balanced diet, limiting salt and sugar intake, and avoiding tobacco and excessive alcohol consumption are key preventive strategies.

### What is known about this topic

- Non-communicable diseases mortality: non-communicable diseases including diabetes and hypertension, accounted for 74% of global deaths in 2022;
- Hypertension-diabetes link: hypertension and diabetes frequently coexist, increasing cardiovascular and other health risks;
- Undiagnosed diabetes: significant prevalence of undiagnosed diabetes exists among patients with hypertension, as seen in studies from Uganda and Nigeria.

### What this study adds

- Undiagnosed diabetes: 5% had undiagnosed diabetes; 19.5% were pre-diabetes;
- Risk factors: key risk factors included age, BMI > 25 kg/m^2^, and marital status;
- Gender and education: higher rates in women and those with lower education.

## References

[1] Sun H, Saeedi P, Karuranga S, Pinkepank M, Ogurtsova K, Duncan BB (2022). IDF Diabetes Atlas: Global, regional and country-level diabetes prevalence estimates for 2021 and projections for 2045. Diabetes Res Clin Pract..

[2] Hendriks ME, Wit FWNM, Roos MTL, Brewster LM, Akande TM, de Beer IH (2012). Hypertension in sub-Saharan Africa: cross-sectional surveys in four rural and urban communities. PLoS One.

[3] World Health Organization (2013). A global brief on hypertension: world health day.

[4] Parati G, Kilama MO, Faini A, Facelli E, Ochen K, Opira C (2010). A new solar-powered blood pressure measuring device for low-resource settings. Hypertension.

[5] Hall V, Thomsen R, Henriksen O, Lohse N (2011). Diabetes in sub-Saharan Africa 1999-2011: epidemiology and public health implications. A systematic review. BMC Public Health..

[6] World Health Organization (WHO) (2023). Global Report on Hypertension. WHO;.

[7] Chatterjee R, Narayan KMV, Lipscomb J, Jackson SL, Long Q, Zhu M (2013). Screening for diabetes and prediabetes should be cost-saving in patients at high risk. Diabetes Care.

[8] Statistics BO (2015). Kenya STEPwise Survey for Non-Communicable Diseases Risk Factors Report 2015.

[9] Ayah R, Joshi MD, Wanjiru R, Njau EK, Fredrick Otieno C, Njeru EK (2013). A population-based survey of prevalence of diabetes and correlates in an urban slum community in Nairobi. Kenya. BMC Public Health..

[10] Jenson A, Omar AL, Omar MA, Rishad AS, Khoshnood K (2011). Assessment of hypertension control in a district of Mombasa, Kenya. Glob Public Health.

[11] Agyei-Mensah S, De-Graft Aikins A (2010). Epidemiological transition and the double burden of disease in Accra, Ghana. J Urban Health.

[12] Mutebi E, Nakwagala FN, Nambuya A, Otim M (2012). Undiagnosed diabetes mellitus and impaired glucose tolerance among patients with hypertension in Mulago Hospital, Kampala, Uganda. Afr J Diabetes Med.

[13] US Preventive Services Task Force (2008). Screening for type 2 diabetes mellitus in adults: US Preventive Services Task Force recommendation statement. Ann Intern Med.

[14] Agardh EE, Sidorchuk A, Hallqvist J, Ljung R, Peterson S, Moradi T (2011). Burden of type 2 diabetes attributed to lower educational levels in Sweden. Popul Health Popul Health Metr.

[15] Lüders S, Hammersen F, Kulschewski A, Venneklaas U, Züchner C, Gansz A (2005). Diagnosis of impaired glucose tolerance in patients with hypertension in daily clinical practice. Int J Clin Pract.

[16] Shang X, Li J, Tao Q, Li J, Li X, Zhang L (2013). Educational level, obesity and incidence of diabetes among Chinese adult men and women aged 18-59 years old: an 11-year follow-up study. PLoS One.

[17] Kidney RSM, Peacock JM, Smith SA (2014). Blood glucose screening rates among minnesota adults with hypertension, behavioral risk factor surveillance system 2011. Prev Chronic Dis..

[18] Iloh GU, Uchenna NR, Obiegbu NP (2013). Risk factors of pre-diabetes among adult Nigerians with essential hypertension in a resource-constrained setting of a primary care clinic in Eastern Nigeria. Am J Heal Res.

[19] Agardh E, Allebeck P, Hallqvist J, Moradi T, Sidorchuk A (2011). Type 2 diabetes incidence and socio-economic position: a systematic review and meta-analysis. Int J Epidemiol.

[20] Kautzky-Willer A, Harreiter J, Pacini G (2016). Sex and gender differences in risk, pathophysiology, and complications of type 2 diabetes mellitus. Endocr Rev.

[21] Tajdar D, Lühmann D, Fertmann R, Steinberg T, van den Bussche H, Scherer M (2021). Low health literacy is associated with higher risk of type 2 diabetes: a cross-sectional study in Germany. BMC Public Health.

[22] Schillinger D, Grumbach K, Piette J, Wang F, Osmond D, Daher C (2002). Association of health literacy with diabetes outcomes. JAMA.

[23] Gordon B (2022). The impact of food insecurity on glycemic control in individuals with diabetes. BioMed.

[24] DeFronzo RA, Ferrannini E, Zimmet P, Alberti G (2015). International Textbook of Diabetes Mellitus, 4^th^ ed.

[25] Hu FB (2011). Globalization of diabetes: the role of diet, lifestyle, and genes. Diabetes Care.

[26] Ginter E, Simko V (2012). Type 2 diabetes mellitus, pandemic in 21^st^ century. Adv Exp Med Biol.

[27] Vazquez G, Duval S, Jacobs DR, Silventoinen K (2007). Comparison of body mass index, waist circumference, and waist/hip ratio in predicting incident diabetes: a meta-analysis. Epidemiol Rev.

[28] Muraki I, Imamura F, Manson JE, Hu FB, Willett WC, van Dam RM (2013). Fruit consumption and risk of type 2 diabetes: results from three prospective longitudinal cohort studies. BMJ..

[29] Micha R, Mozaffarian D (2010). Saturated fat and cardiometabolic risk factors, coronary heart disease, stroke, and diabetes: a fresh look at the evidence. Lipids.

[30] Imamura F, Micha R, Wu JH, de Oliveira Otto MC, Otite FO (2016). Effects of saturated fat, polyunsaturated fat, monounsaturated fat, and carbohydrate on glucose-insulin homeostasis: a systematic review and meta-analysis of randomised controlled feeding trials. PLoS Med.

